# Supplementary material for: Viability discrimination of bacterial microbiomes in home kitchen dish sponges using propidium monoazide treatment
Source: Environ Microbiol Rep. 2024 Oct 23;16(5):e70006. doi: 10.1111/1758-2229.70006 (PMC11497490; doi:10.1111/1758-2229.70006)
Supplement: Supplementary file 1 — Figure S1. The biomass of bacterial communities within each sponge ranged from 0.25 to 5.18 log CFU/sponge. Sponge bacterial community biomass was assessed via qPCR targeting the 16S rRNA gene to estimate the total number of 16S rRNA gene copies present for each sponge. The standard curve was generated from a 1:10 serially diluted Escherichia coli K12 culture. Open, black circles represent the enumerated E. coli culture, and the dashed line represents the standard curve. Points of various shapes and colours represent each sponge's estimated biomass level in log colony‐forming units (CFU) per sponge. Table S1. Unique taxa identified and alpha diversity parameters of sponge bacterial communities according to propidium monoazide (PMA) treatment. [file EMI4-16-e70006-s001.docx]

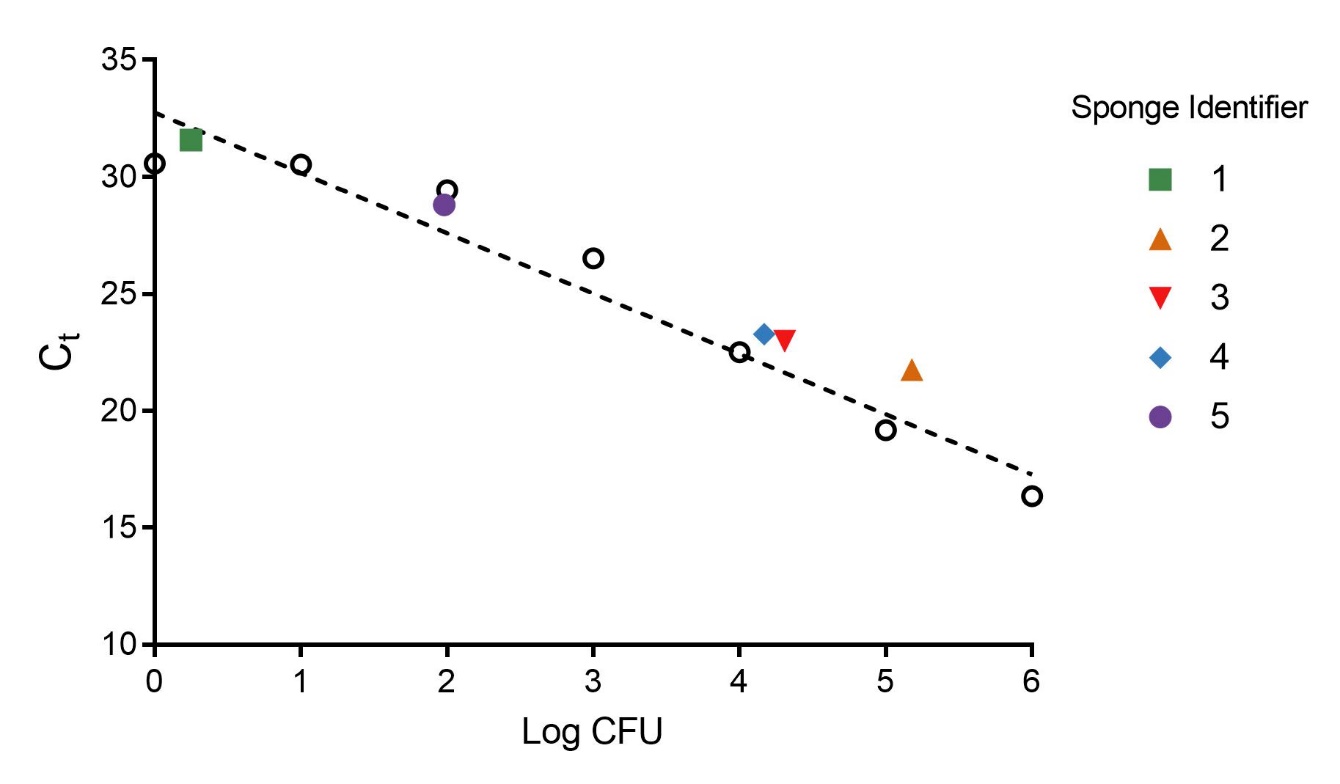


## Figure S1. The biomass of bacterial communities within each sponge ranged from 0.25 to 5.18 log CFU/sponge.

Sponge bacterial community biomass was assessed via qPCR targeting the 16s rRNA gene to estimate the total number of 16S rRNA gene copies present for each sponge. The standard curve was generated from a 1:10 serially diluted *Escherichia* *coli* K12 culture. Open, black circles represent the enumerated *E. coli* culture, and the dashed line represents the standard curve. Points of various shapes and colors represent each sponge's estimated biomass level in log colony-forming units (CFU) per sponge.

## Table S1. Unique taxa identified and alpha diversity parameters*^a^* of sponge bacterial communities according to propidium monoazide (PMA) treatment.

| **Sponge Identifier** | **PMA Treatment** | **Unique Taxonomic Identifications**  (Mean ± SD) | **Chao1 Index**  (Mean ± SD*^c^*) | **Shannon’s Diversity Index**  (Mean ± SD) | **Simpson’s Index**  (Mean ± SD) | **Simpson’s Reciprocal Index**  (Mean ± SD) |
| --- | --- | --- | --- | --- | --- | --- |
| 1 | No | 29 ± 16 | 49 ± 44 | 2.36 ± 0.06 | 0.86 ± 0.01 | 7.06 ± 0.42 |
| 1 *^b^* | Yes | 34 ± 0 | 34 ± 0 | 2.99 ± 0.00 | 0.92 ± 0.00 | 12.03 ± 0.00 |
| 2 | No | 28 ± 18 | 51 ± 51 | 1.29 ± 0.39 | 0.54 ± 0.17 | 2.32 ± 0.85 |
| 2 | Yes | 37 ± 5 | 37 ± 5 | 1.24 ± 0.45 | 0.57 ± 0.27 | 2.9 ± 1.79 |
| 3 | No | 28 ± 25 | 60 ± 56 | 1.71 ± 0.02 | 0.69 ± 0.00 | 3.2 ± 0.01 |
| 3 *^b^* | Yes | 32 ± 0 | 44 ± 0 | 2.69 ± 0.00 | 0.87 ± 0.00 | 7.68 ± 0.00 |
| 4 | No | 25 ± 8 | 51 ± 25 | 1.77 ± 0.30 | 0.69 ± 0.11 | 3.47 ± 1.32 |
| 4 | Yes | 37 ± 1 | 60 ± 34 | 1.95 ± 0.16 | 0.74 ± 0.06 | 4.04 ± 1.03 |
| 5 | No | 16 ± 1 | 30 ± 6 | 1.38 ± 0.25 | 0.53 ± 0.07 | 2.14 ± 0.33 |
| 5 *^b^* | Yes | 8 ± 0 | 11 ± 0 | 1.76 ± 0.00 | 0.78 ± 0.00 | 4.49 ± 0.00 |
| SD, Standard deviation  *^a^*, Alpha diversity parameter computations were conducted using the relative abundances or the number of reads assigned to each taxon averaged across replicates unless otherwise indicated.  *^b^*, Alpha diversity parameters were computed for one replicate.  *^c^*, Represents standard deviation of Chao1 index values across replicate samples. | | | | | | |
